# TNFAIP3 alleviates cerebral ischemia-reperfusion injury by inhibiting M1 microglia polarization via deubiquitination of RACK1

**DOI:** 10.1371/journal.pone.0337601

**Published:** 2025-11-26

**Authors:** Wenya Bai, Shixuan Liu, Guilin Zhou, Xuelian Li, Huan Jiang, Jianlin Shao, Junchao Zhu

**Affiliations:** 1 Department of Anesthesiology, The First Affiliated Hospital of Kunming Medical University, Kunming, Yunnan, P. R. China; 2 Department of Anesthesiology, The Shengjing Hospital of China Medical University, Shenyang, Liaoning, P. R. China; Lund University, SWEDEN

## Abstract

**Background:**

Microglia polarization plays a crucial role in the progression of cerebral ischemia-reperfusion injury (CIRI), but the mechanisms remain largely undefined. The preset study aimed to investigate the mechanism of microglia polarization following CIRI.

**Methods:**

CIRI was modeled in C57BL/6J mice through middle cerebral artery occlusion-reperfusion and in BV2 cells via oxygen and glucose deprivation/reoxygenation. Reverse transcription-quantitative PCR, western blotting, flow cytometry and fluorescence staining were used to detect the expression levels of key proteins associated with microglia polarization, as well as the expression of TNFAIP3 and RACK1. The interaction between TNFAIP3 and RACK1 was verified by co-immunoprecipitation. TNFAIP3 or RACK1 gene interference (overexpression and/or silencing) was employed to examine the role of the TNFAIP3/RACK1 axis in microglia polarization following CIRI.

**Results:**

The results revealed that Arg-1 expression decreased, inducible nitric oxide synthase expression increased and TNFAIP3 was upregulated 24 h after CIRI. Furthermore, TNFAIP3 interacted with RACK1 to deubiquitinate and increase the expression of RACK1. These results indicate that knocking down either TNFAIP3 or RACK1 promotes microglia M1 polarization, and overexpression of RACK1 can promote microglia M2 polarization. RACK1 exerts its neuroprotective effects through NF-κB, as demonstrated by the use of NF-κB inhibitors.

**Conclusion:**

The present findings indicate that TNFAIP3 inhibits M1 microglial polarization via deubiquitination of RACK1 after CIRI, RACK1 exerts its effects through NF-κB.

## Introduction

Stroke, also known as ‘brain attack’, is an event in which blood supply fails to be delivered to the brain because of a sudden rupture or blockage of the cerebral blood vessels. It is mainly divided into two types: Ischemic stroke and hemorrhagic stroke [1,2]. Stroke is the second leading cause of death worldwide and the third leading cause of death and disability [3]. Stroke affects 15 million people worldwide each year, resulting in 5 million deaths and leaving another 5 million with permanent physical disabilities. Ischemic stroke is a major cause of permanent physical disability [4]. The China Stroke Prevention and Treatment Report 2021 identify stroke as a major threat to the physical and mental health of Chinese citizens, being the primary cause of mortality and disability among adults in the country. By 2024, the global economic burden of stroke will exceed $890 billion, accounting for 0.66% of global GDP [5]. It is marked by notable morbidity, recurrence, disability, mortality, and economic burden. With the acceleration of population aging and urbanization, exposure to risk factors for cerebrovascular diseases has increased, resulting in an augmented burden of cerebrovascular diseases [6]. Ischemic stroke, the most prevalent form of stroke, constitutes 87% of all stroke cases. After an ischemic stroke, neurons in the core ischemic area have insufficient glucose and oxygen supply and experience an imbalance in energy metabolism, which may ultimately lead to irreversible neuron damage [7]. Effective acute ischemic stroke treatment focuses on preserving the ischemic penumbra by promptly restoring blood flow through thrombolysis or endovascular therapy. However, because of the short treatment time, only 5% of patients benefit from treatment. Thrombolysis can result in cerebral ischemia-reperfusion injury (CIRI), causing additional tissue damage and potentially irreversible effects such as edema and hemorrhage. CIRI is caused by the integrated action of multiple steps and is related to the secondary brain injury caused by a variety of factors through multiple pathways [8]. Mitigation of CIRI is a research hotspot, and elucidating the mechanism of the onset of CIRI may provide new ideas and targets for its treatment. The core mechanism of CIRI involves an inflammatory cascade mediated by inflammatory cells and cytokines. Reperfusion exacerbates tissue damage by eliciting the release of a substantial quantity of inflammatory mediators [9].

Previous studies have demonstrated the important role of microglia-mediated inflammatory responses in the development of CIRI [10–12]. Microglia, a prevalent intrinsic immune cell type in the central nervous system, continuously monitor the intracerebral environment. Once pathogen invasion or tissue damage is identified, microglia immediately migrate to the corresponding sites to remove the pathogens or participate in tissue repair and remodeling by phagocytosis or secreting inflammatory factors. Microglia exhibit remarkable plasticity and can differentiate into pro-inflammatory (M1) or anti-inflammatory (M2) phenotypes when stimulated [13]. M1 microglia exhibit elevated CD86 and inducible nitric oxide synthase (iNOS) levels, releasing pro-inflammatory cytokines such as tumor necrosis factor (TNF)-α, interleukin (IL)-18, and IL-6, potentially leading to brain tissue damage and neuronal apoptosis [14]. M2 microglia exhibit elevated Arg-1 and CD163 levels and release anti-inflammatory cytokines such as IL-4, IL-10, and TGF-β, which mitigate inflammatory responses and decrease brain tissue damage [15]. Previous research indicates that quercetin alleviates CIRI by enhancing M2 polarization of microglia/macrophages through modulation of the PI3K/Akt/NF-κB signaling pathway [16]. A previous study demonstrated that curcumin preconditioning improved the ability of OM-MSCs to mitigate neuroinflammation by influencing microglia polarization through the upregulation of microRNA-423-5p. This intervention effectively reduced PANoptotic neuronal death caused by CIRI [17]. Thus, although CIRI induces dynamic phenotypic changes of microglia, it promotes M2 microglia polarization, which may provide novel diagnostic and therapeutic bases for the treatment and prevention of ischemic stroke.

TNFAIP3 (Tumor Necrosis Factor Alpha-Induced Protein 3, A20) exhibits deubiquitinating enzyme activity in the N-terminal OTU domain, allowing it to hydrolyze K63-linked polyubiquitin chains and thereby modulate signal transduction pathways. At the C-terminal end, TNFAIP3 contains seven consecutive zinc finger domains that act as E3 ubiquitin ligases. These domains promote the assembly of K48-linked polyubiquitin chains, aiding in substrate protein recognition by the proteasome for degradation [18]. TNFAIP3, a ubiquitin-editing enzyme, has been shown to inhibit NF-κB signaling activation through various mechanisms [19]. Cells deficient in TNFAIP3 demonstrate heightened sensitivity to inflammatory cytokine stimulation, culminating in prolonged NF-κB activation [20]. Targeted TNFAIP3 gene knockout in the central nervous system triggers abnormal activation of heterotropic inflammatory cells and increases inflammatory mediator expression in the murine brain [21]. TNFAIP3 negatively regulates NLRP3 inflammasome expression by inhibiting the NF-κB signaling pathway, which in turn suppresses pyroptosis [22]. Previous research has also shown that TNFAIP3 mitigates the inflammatory response and programmed necrosis after traumatic brain injury by inhibiting NLRP3 inflammasome expression [23]. Further research is needed to determine the role of TNFAIP3 in microglia polarization after CIRI. The aim of the present study was to elucidate whether TNFAIP3 can reduce CIRI by regulating microglia polarization and its specific molecular mechanism.

## Materials and methods

### Animals

Adult male C57BL/6J mice, weighing 20 ± 2 g, were sourced from the Experimental Animal Centre at Kunming Medical University. Before the experiment, mice were kept for 1 week in an SPF-level barrier environment with controlled temperature and pressure, and a 12-h light cycle. The present study adheres to the National Institutes of Health (NIH) guidelines for laboratory animal care and use, aiming to minimize the number of mice used and alleviate their suffering during experiments. The Experimental Animal Ethics Committee of Kunming Medical University approved all animal experiments conducted in the current study (approval no. kmmu20240468). The mice were fasted for 3 h and then anesthetized with isoflurane (AErrane; Baxter Healthcare Corporation) by inhalation (2.5−3% for induction and 2–2.5% for maintenance, with oxygen) at a rate of 1.5 l/min. During the entire procedure, the rats were plated on a thermal blanket to maintain body temperature at 36.5 ± 0.5°C with a regulated heating pad till recovery from anesthesia. Euthanasia of mice with cervical dislocation after anesthesia with sodium pentobarbital (120 mg/kg). This study was strictly adhere to the ARRIVE guidelines.

### Animal model of CIRI

The middle cerebral artery occlusion-reperfusion (MCAO/R) model was developed using a modified Zea-Longa intraluminal filament technique. After anesthetizing and securing the mice, a midline neck incision was made to expose the right common carotid artery (CCA), external carotid artery (ECA) and internal carotid artery (ICA). The proximal CCA and distal ECA were ligated, and the ECA was transected. A poly-lysine-coated nylon filament was inserted into the ECA stump through the ICA to occlude the MCA. The incision above the ECA was ligated and the wound was closed. The filament was withdrawn after 30 min. All animals were provided with meticulous postoperative care with adequate water and food. Mice were divided into a sham surgery group (Sham, n = 3) and an MCAO/R model group (MCAO/R, n = 15). The MCAO/R group was further divided into MCAO/R-3, MCAO/R-6, MCAO/R-12, MCAO/R-24, and MCAO/R-48 groups (n = 3) based on the time points of 3 h, 6 h, 12 h, 24 h, and 48 h after reperfusion.

### Reverse transcription-quantitative PCR (RT-qPCR)

Under sterile, enzyme-inactivated conditions, brain tissue from the ischemic penumbra on the reperfusion side was homogenized to extract total RNA, which was then reverse-transcribed into cDNA and amplified via PCR. The standard curve exhibited R2 ≥ 0.99. A single peak in the melting curve and a single band on agarose gel confirmed primer specificity without primer-dimer or nonspecific products. PCR cycle reaction conditions: 95°C:10 min; 95°C: 15 s; 60°C: 30 s; 40–45 cycles. The qPCR reaction determined the Ct value for each sample, with relative mRNA expression calculated using β-actin as an endogenous control. The study utilized specific primer pairs (5’-3’). TNFAIP3-forward: CGCTGTTCCACTTGTTAA and TNFAIP3-reverse: TTCCTTCATCTCATTCTCAG. β-actin -forward: CTGGAGAAGAGCTATGAG and β-actin -reverse: GATGGAATTGAATGTAGTTTC.

### Western blotting

Protein extraction using RIPA buffer (P0013B, Beyotime,) from brain tissue in the ischemic penumbra on the IR side involved homogenization and centrifugation, followed by total protein quantification with ultraviolet spectrophotometer. Proteins were then separated using 10% SDS-PAGE and transferred to a PVDF membrane for antibody binding and analysis. The membrane was blocked with 5% skimmed milk. The membranes were incubated overnight at 4°C with primary antibodies targeting β-actin (1:5,000; Proteintech Group, Inc.), TNFAIP3 (1:500; Proteintech Group, Inc.), RACK1 (1:1,000; Abcam), Arg-1 (1:500; Affinity Biosciences), iNOS (1:2,000; Proteintech Group, Inc.), CD206 (1:5,000; Proteintech Group, Inc.), CD16/32 (1:500; Affinity Biosciences), Goat Anti Mouse IgG -HRP (1:4000, M21001L, Abmart), and Goat Anti Rabbit IgG-HRP (1:4000, M21002L, Abmart).

### Cell culture

Mouse BV2 microglial cells from the China Center for Type Culture Collection were maintained in DMEM supplemented with 10% fetal bovine serum and 1% penicillin-streptomycin. The cells were incubated at 37°C in a humidified 5% CO_2_ environment, with medium changes every 2 days.

### Oxygen-glucose deprivation/reoxygenation (OGD/R)

Cells were removed from the incubator, and the medium was replaced with glucose-free DMEM. Cells were then incubated at 37°C in an anaerobic environment (containing 94% N_2_, 5% CO_2_, and 1% O_2_) for 2 h. Following anaerobic treatment, the medium was exchanged for normal medium, and cells were cultured under standard conditions (37°C, containing 5% CO_2_, and 95% air) for 12 h.

### Cell transfection

Lentivirus, with a known titer (1.5x10^8^ TU/mL), was diluted in normal culture medium to achieve a multiplicity of infection of 20. Polybrene (final concentration 5 µg/mL) was added to enhance transduction efficiency. The cell monolayer in the culture flask was incubated with this lentiviral-containing medium for 24–48 h. Following infection, the medium was replaced with fresh growth medium containing puromycin to select for stably transduced cells. Selection was maintained for 7 days, or until all non-transduced control cells were eliminated. Cell populations were then expanded through passaging for subsequent experiments. Treatment with ammonium pyrrolidinedithiocarbamate (PDTC) was used to inhibit the activity of NF-κB.

### Co-immunoprecipitation (Co-IP)

The cells were treated with pre-chilled lysis buffer, lysed for 30 min, and then centrifuged to collect the supernatant. An equal volume of protein A/G agarose beads was added and incubated for 1 h, followed by the addition of 2 μL of TNFAIP3 antibody (IP group, 1:500, Proteintech) or IgG (IgG group, 1:4000, Abmart), mixed, and incubated overnight. Subsequently, 20 μL of protein A/G agarose beads was added and incubated for 2 h to capture the antibody-antigen complex. Wash and centrifuge to collect the precipitate, then resuspend the beads in 1 × Laemmli SDS-PAGE loading buffer. All the above experimental steps were performed at 4°C. Subsequently, the complex was incubated at 95°C in a metal bath for 10 min for elution and denaturation. The denatured samples were adjusted to a protein loading amount of 60 μg for Western blot detection and mass spectrometric analysis.

### Immunofluorescence (IF)

BV2 microglial cells were seeded on coverslips, fixed in 4% PFA for 20 min and permeabilized with 0.5% Triton X-100 for 5 min at room temperature. Subsequently, cells were incubated with 3% BSA for 30 min at 37°C to block non-specific binding, and then exposed to primary antibodies at 4°C overnight, including anti-CD206 rabbit polyclonal antibody (1:100; cat. no. DF4149; Affinity Biosciences) and anti-CD16/32 (1:500; cat. no. ab223200; Abcam). After rewarming and rinsing, cells were incubated with Goat Anti-Rabbit IgG (H + L) (1:500; cat. no. A11008, Thermo Fisher) at 37°C, 1 h, in the darkness. An investigator, unaware of the experimental design, used ImageJ software (v 1.53j, NIH) to quantify the positive signals.

### Identification of TNFAIP3 ubiquitinated substrates

After TNFAIP3 knockdown, cells underwent a 6-h treatment with 40 μM MG-132, followed by OGD/R treatment, and RACK1 protein expression was analyzed using western blotting. Following TNFAIP3 knockdown, cells were treated with cycloheximide (CHX) at various time points, and RACK1 protein expression was analyzed via western blotting.

### Flow cytometry (FCM)

The cell suspension of each group was divided into two parts. After centrifuging at room temperature, the supernatant was discarded, and the cells were resuspended, washed with 2 mL PBS and centrifuged again. This procedure was repeated twice. Cells were then resuspended with M1 (CD16/32) or M2 (CD206) markers based on grouping, and then incubated in a CO_2_ incubator in the dark for 30 min. Subsequently, each tube was washed by adding 200 mL PBS. Cells were then resuspended and loaded into a flow cytometer (VBR, Novocyte advanteon) for detection.

### Statistical analysis

All experimental data were performed with at least three biological replicates and technical replicates. All statistical analyses were performed with the SPSS software (version 22.0, IBM Corp). Continuous variables are represented as the mean ± standard deviation (SD). Data were analyzed by one-way ANOVA followed by Tukey’s post hoc test. Statistical significance was considered at P < 0.05.

## Results

### Expression of TNFAIP3 in the mouse MACO/R model

**Fig 1:**
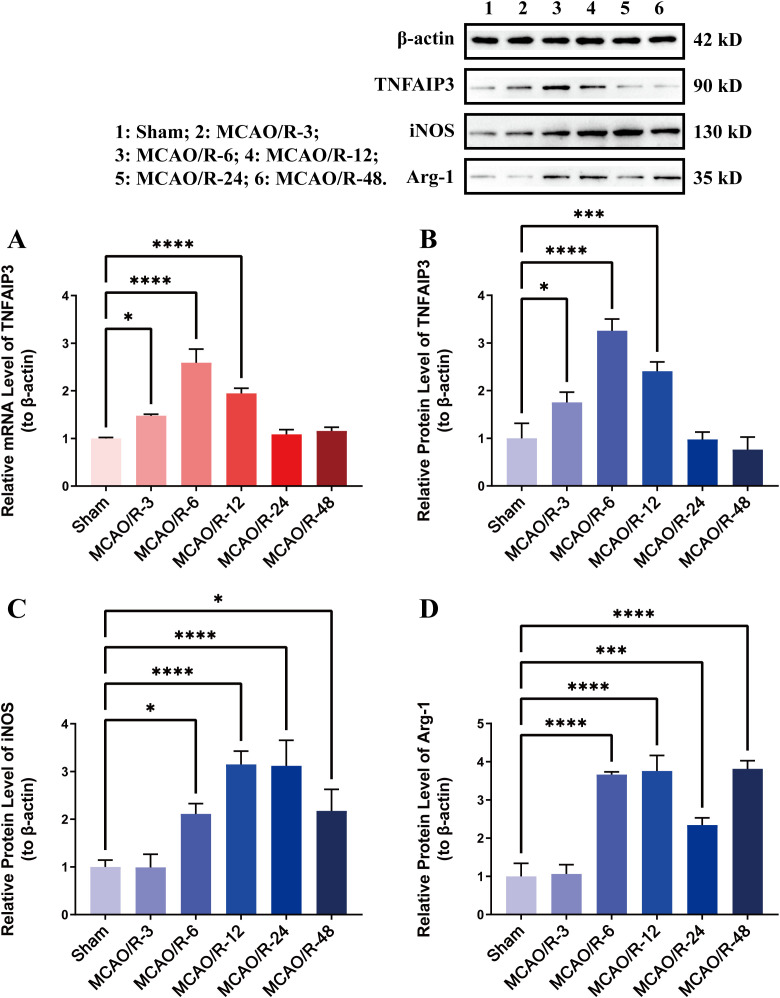
TNFAIP3 expression and microglia polarization at different time points in the MCAO/R model. **(A)** Expression levels of TNFAIP3 mRNA; **(B)** Quantitative analysis of TNFAIP3 protein expression; **(C)** Quantitative analysis of iNOS protein levels; **(D)** Quantitative analysis of Arg-1 protein levels. Data are presented as the mean ± SD from replicate experiments (n = 3). *P < 0.05; **P < 0.01; ***P < 0.001. The endpoints of the line segment indicate the comparison between the two groups under consideration.

TNFAIP3 expression in mouse I/R brain tissue was quantified at specified time points (3, 6, 12, 24 and 48 h post-reperfusion). The findings showed a notable rise in TNFAIP3 expression relative to the Sham group, with a peak at 6 hours after CIRI (Fig 1A, 1B). These results demonstrate that TNFAIP3 was involved in the development of CIRI.

### Microglial M1 polarization was induced by CIRI

Our study examined microglial polarization at different time points after CIRI, revealing a significant decrease in Arg-1 expression and an increase in iNOS expression at 6, 12, 24, and 48 h after CIRI, with the peak expression at the time points of 24 h after CIRI. Then, we chose the time point of 24 h in the following experiments (Fig 1C, 1D). These results demonstrate that M1 polarization of microglia promotes the development of CIRI.

### RACK1 serves as a substrate for the deubiquitinating activity of TNFAIP3

**Fig 2:**
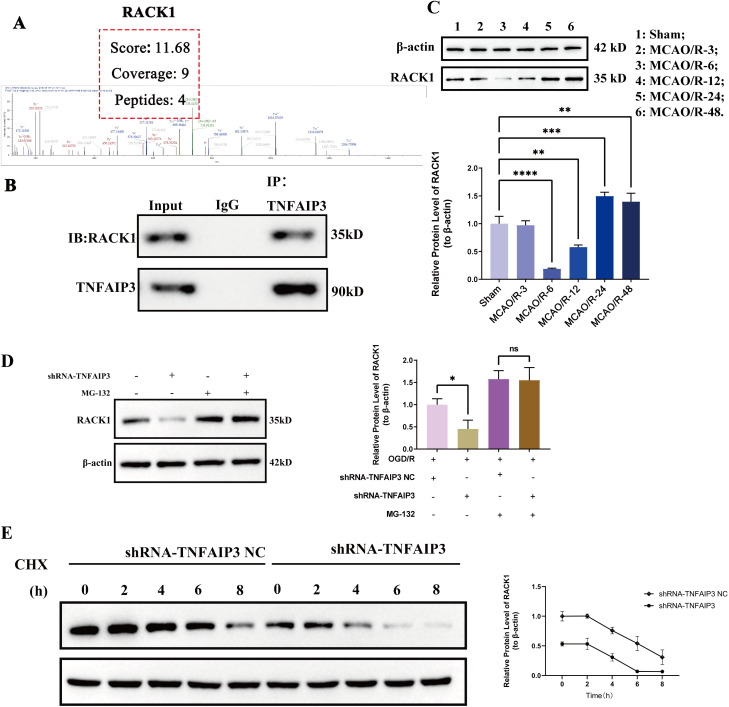
RACK1 is a ubiquitinated substrate of TNFAIP3. **(A)** Mass spectrometry data identifying RACK1 among the TNFAIP3-interacting proteins; **(B)** Co-immunoprecipitation (Co-IP) assay confirming the interaction between TNFAIP3 and RACK1; **(C)** Quantitative analysis of RACK1 protein levels; **(D)** Quantitative analysis of RACK1 protein levels in TNFAIP3-knockdown cells following MG132-mediated stimulation; **(E)** RACK1 degradation in TNFAIP3-knockdown cells exposed to cycloheximide (CHX) at the indicated time points. Data are presented as the mean ± SD from replicate experiments (n = 3). *P < 0.05; **P < 0.01; ***P < 0.001. The endpoints of the line segment indicate the comparison between the two groups under consideration.

To understand the molecular mechanisms through which TNFAIP3 inhibits microglia polarization in OGD/R-injured microglial cells, a co-IP assay was conducted using an anti-TNFAIP3 antibody to isolate interacting proteins. Based on the significance score (score), the proportion of amino acids covered by the measured peptide segments (coverage), and the number of peptide segments that only matched TNFAIP3 (peptides) obtained from mass spectrometry analysis were comprehensively analyzed, RACK1 was selected (Fig 2A). The results of co-IP assay verified the interaction between TNFAIP3 and RACK1, indicating a possible interaction between RACK1 and TNFAIP3 (Fig 2B). In the MACO/R model, the expression level of RACK1 first decreased and then significantly increased (Fig 2C).

Considering that TNFAIP3 is an enzyme that is associated with ubiquitination, it was hypothesized that TNFAIP3 may deubiquitinate RACK1. The impact of TNFAIP3 expression changes on RACK1 protein levels was assessed, with and without the proteasome inhibitor MG-132 to stabilize RACK1. Western blot analysis indicated that MG-132 treatment nullified the impact of TNFAIP3 on RACK1 expression in BV2 cells (Fig 2D). The current study used CHX to inhibit protein synthesis and evaluate the stability of RACK1 in cells. The results showed a significant reduction in the half-life of RACK1 expression in TNFAIP3-knockdown neurons compared to parental cells within the same time interval (Fig 2E). These findings suggest that TNFAIP3 enhances RACK1 expression by modulating its ubiquitination.

### Downregulation of TNFAIP3 aggravated OGD/R injury by decreasing the expression of RACK1

**Fig 3:**
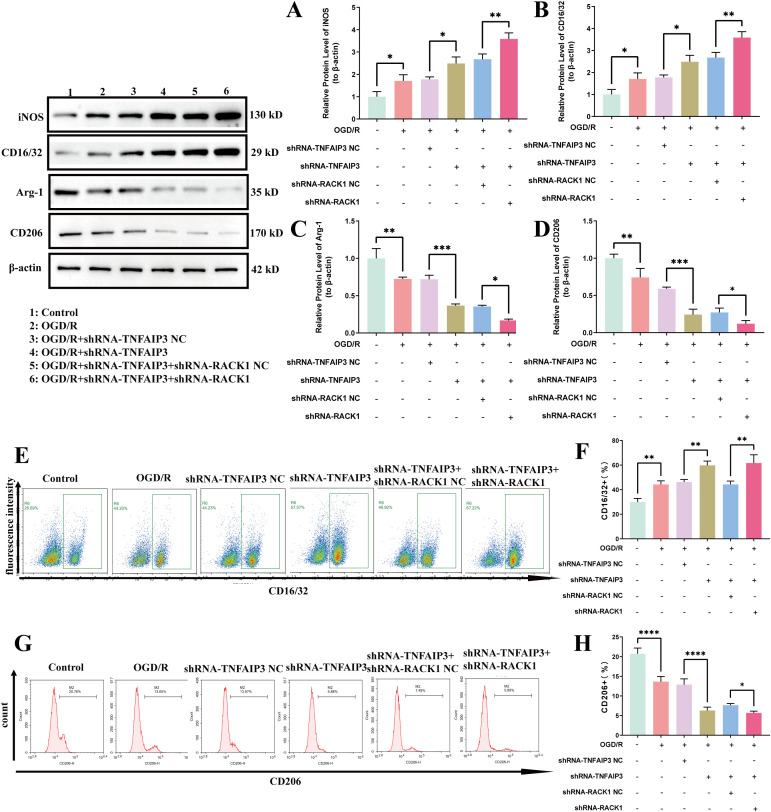
The effect of downregulating TNFAIP3 and/or RACK1 expression on microglia polarization. **(A)** Quantitative analysis of iNOS protein levels; **(B)** Quantitative analysis of CD16/32 protein levels; **(C)** Quantitative analysis of Arg-1 protein levels; **(D)** Quantitative analysis of CD206 protein levels. **(E-F)** Flow cytometry (FCM) analysis of the proportion of CD16/32 + cells in the different experimental groups; **(G-H)** FCM analysis of the proportion of CD206 + cells in the different experimental groups. Data are presented as the mean ± SD from replicate experiments (n = 3). *P < 0.05; **P < 0.01; ***P < 0.001. The endpoints of the line segment indicate the comparison between the two groups under consideration.

To further examine the role of the TNFAIP3/RACK1 axis in regulating CIRI, an OGD/R model was constructed after transfecting BV2 cells with downregulated TNFAIP3 and RACK1 lentivirus vectors or their negative controls. Microglia polarization was estimated. We transfected shRNA-TNFAIP3 and its negative controls into BV2 cells, followed by OGD/R stimulation. The results indicated that reducing TNFAIP3 expression enhanced microglia M1 polarization, as evidenced by elevated iNOS and CD16/32 protein levels, alongside decreased Arg-1 and CD206 protein levels. Rescue assays were conducted to determine if RACK1 downregulation could reverse the effects of TNFAIP3 knockdown. The results demonstrated a significant reduction in Arg-1 and CD206 expression levels in the OGD/R + shRNA-TNFAIP3 + shRNA-RACK1 group (sh-both) compared with the OGD/R + shRNA-TNFAIP3 + shRNA-RACK1 NC group (sh-both-NC). In the sh-both group, the levels of iNOS and CD16/32 were notably higher than those in the sh-both-NC group (Fig 3A–3D).

**Fig 4:**
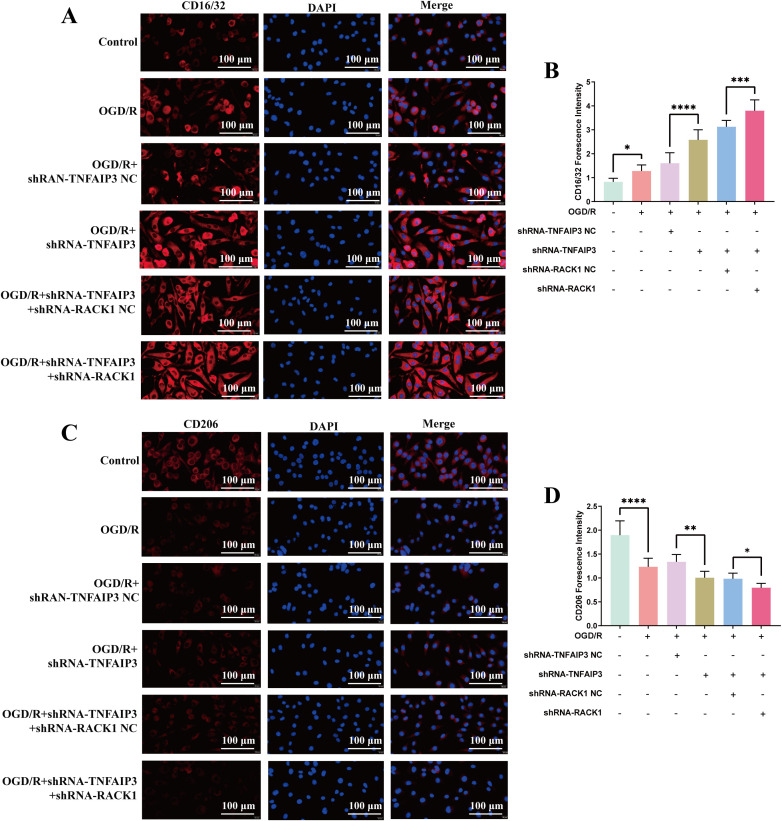
The effect of downregulating TNFAIP3 and/or RACK1 expression on microglia polarization. **(A-B)** Representative images of double immunostaining and quantitative analysis of BV2 microglial M1 polarization, with CD16/32 (red) and DAPI (green); Scale bar = 100 μm. **(C-D)** Representative images of double immunostaining and quantitative analysis of BV2 microglial M1 polarization, with CD206 (red) and DAPI (green); Scale bar = 100 μm. Data are presented as the mean ± SD from replicate experiments (n = 3). *P < 0.05; **P < 0.01; ***P < 0.001. The endpoints of the line segment indicate the comparison between the two groups under consideration.

The FCM experiments also showed that, compared with the sh-both-NC group, the fluorescence intensity of CD16/32 was increased and the fluorescence intensity of CD206 was decreased in the sh-both group (Fig 3E–3H). The IF experiments also showed increased CD16/32 expression and decreased CD206 expression in the sh-both group (Fig 4). These findings indicate that RACK1 downregulation mitigates the worsening of OGD/R injury induced by TNFAIP3 downregulation.

### Upregulating RACK1 promotes microglia M2 polarization

**Fig 5:**
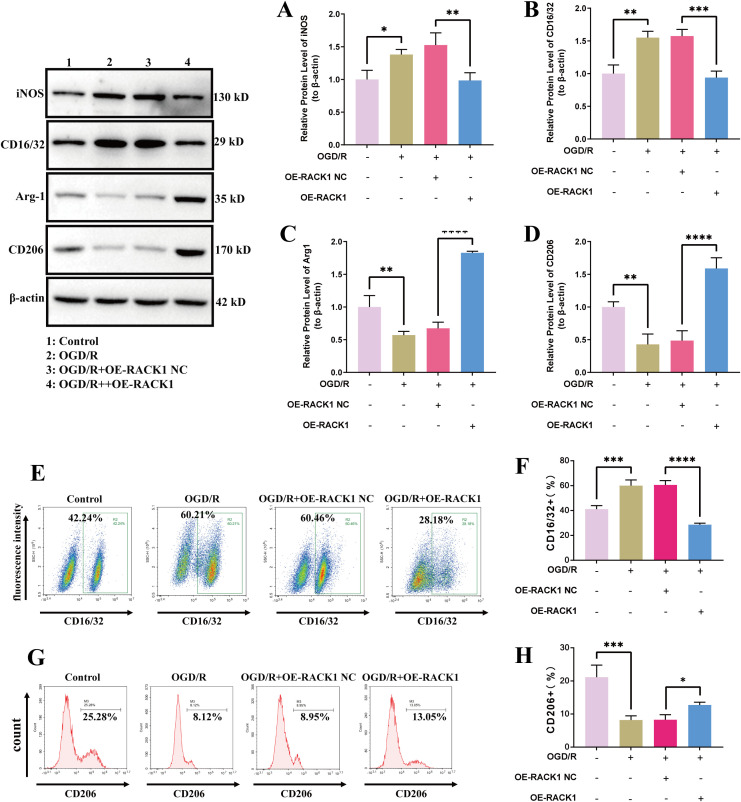
Effects of RACK1 overexpression on microglia polarization. **(A)** Quantitative analysis of iNOS protein levels; **(B)** Quantitative analysis of CD16/32 protein levels; **(C)** Quantitative analysis of Arg-1 protein levels; **(D)** Quantitative analysis of CD206 protein levels. **(E-F)** Flow cytometry (FCM) analysis of the proportion of CD16/32 + cells in the different experimental groups; **(G-H)** FCM analysis of the proportion of CD206 + cells in the different experimental groups. Data are presented as the mean ± SD from replicate experiments (n = 3). *P < 0.05; **P < 0.01; ***P < 0.001. The endpoints of the line segment indicate the comparison between the two groups under consideration.

**Fig 6:**
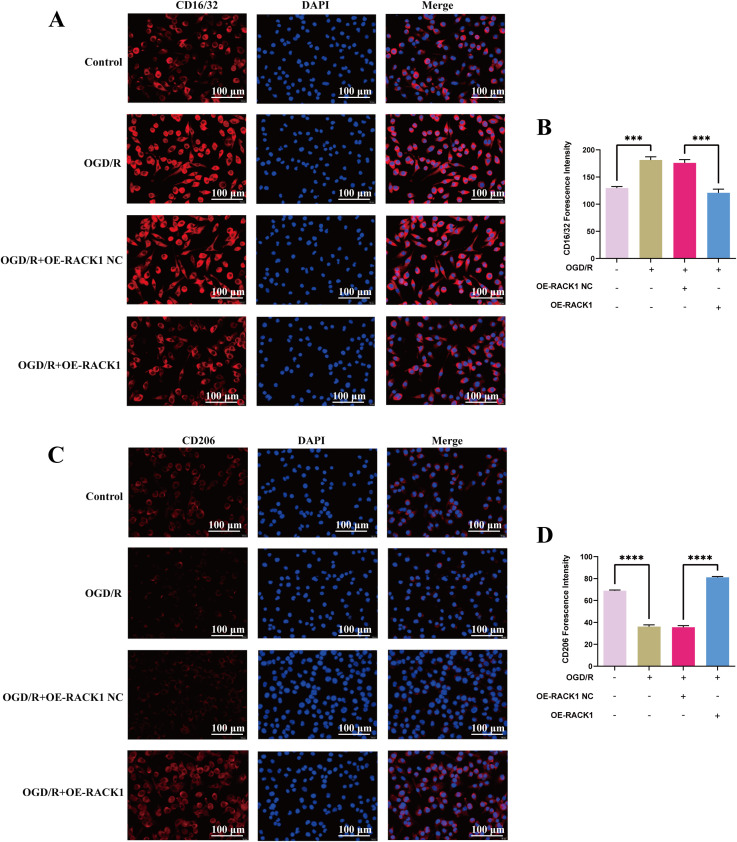
Effects of RACK1 overexpression on microglia polarization. **(A-B)** Representative images of double immunostaining and quantitative analysis of BV2 microglial M1 polarization, with CD16/32 (red) and DAPI (green); Scale bar = 100 μm. **(C-D)** Representative images of double immunostaining and quantitative analysis of BV2 microglial M1 polarization, with CD206 (red) and DAPI (green); Scale bar = 100 μm. Data are presented as the mean ± SD from replicate experiments (n = 3). *P < 0.05; **P < 0.01; ***P < 0.001. The endpoints of the line segment indicate the comparison between the two groups under consideration.

To further investigate the impacts of RACK1 after CIRI, the current study built RACK1 overexpression (OE-RACK1) and negative control (OE-NC) groups. In the OGD/R group, iNOS and CD16/32 expression levels were increased, whereas Arg-1 and CD206 expression levels were reduced compared with those in the control group. Compared with that of the OE-NC group, the expressions of iNOS and CD16/32 showed downregulation in the OE-RACK1 group, while the expressions of Arg-1 and CD206 showed upregulation in the OE-RACK1 group (Fig 5A–5D). The FCM experiments also showed that, compared with the OE-NC group, the fluorescence intensity of CD16/32 was decreased, while the fluorescence intensity of CD206 was increased in the OE-RACK1 group (Fig 5E–5H). The IF experiments also showed decreased CD16/32 expression and increased CD206 expression in the OE-RACK1 group (Fig 6). The findings suggest that enhancing RACK1 expression facilitates microglia M2 polarization and inhibits microglia M1 polarization.

### RACK1 promotes M2 polarization of microglia by inhibiting NF-κB

**Fig 7:**
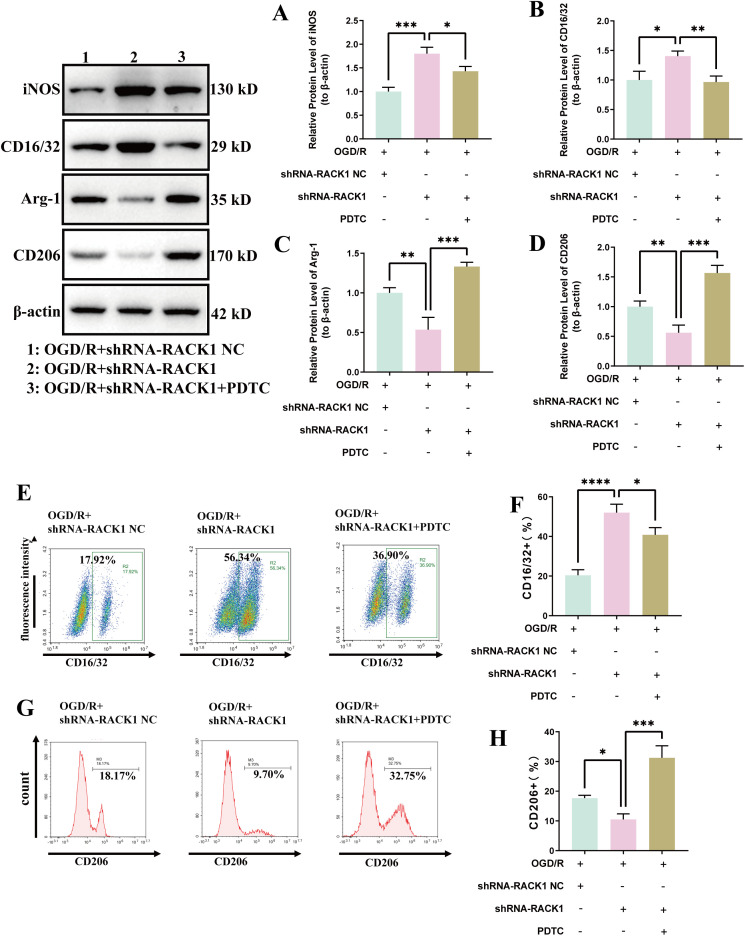
Effects of NF-κB pathway inhibition on microglial polarization. **(A)** Quantitative analysis of iNOS protein levels; **(B)** Quantitative analysis of CD16/32 protein levels; **(C)** Quantitative analysis of Arg-1 protein levels; **(D)** Quantitative analysis of CD206 protein levels. **(E-F)** Flow cytometry (FCM) analysis of the proportion of CD16/32 + cells in the different experimental groups; **(G-H)** FCM analysis of the proportion of CD206 + cells in the different experimental groups. Data are presented as the mean ± SD from replicate experiments (n = 3). *P < 0.05; **P < 0.01; ***P < 0.001. The endpoints of the line segment indicate the comparison between the two groups under consideration.

This study investigated the mechanism of RACK1 using the NF-κB inhibitor PDTC. Results demonstrated that PDTC significantly suppressed shRACK1-induced M1 polarization and promoted M2 polarization in microglia (Fig 7A–7D). Flow cytometry studies also confirmed these findings (Fig 7E–7H). These results indicate that RACK1 promotes M2 polarization in microglia and improves CIRI by inhibiting NF-κB expression.

## Discussion

“Rodent Models of Stroke” [24] identifies five distinct methods for modeling CIRI in animal research. In this study, catheter embolization was employed to generate a MCAO/R model of focal CIRI. Additionally, the OGD/R model is a well-established in vitro model for CIRI research. The combined results from both in vivo and in vitro models demonstrate that CIRI induces microglial polarization towards the M1 phenotype, a process that is linked to the TNFAIP3-RACK1 signaling pathway.

Microglia play a crucial role in the central nervous system’s immune responses, mediating both immunological and inflammatory processes. Their polarization is central to CIRI pathophysiology, as microglia shift between the protective M2 phenotype and the pro-inflammatory M1 phenotype, the latter exacerbating CIRI. Studies have shown that Annexin A1 influences microglial and macrophage polarization through the FPR2/ALX-dependent AMPK-mTOR pathway, thereby offering a protective mechanism against CIRI [25]. Following CIRI, expression of Arg-1, an anti-inflammatory cytokine, is reduced, whereas iNOS, a pro-inflammatory cytokine, is upregulated. The mitochondrial DNA (mtDNA)-STING pathway contributes to M1 polarization of microglia via the IRF3/NF-κB signaling axis, further aggravating CIRI [26]. Quercetin, through modulation of the PI3K/Akt/NF-κB signaling pathway, enhances M2 microglial/macrophage polarization and has been shown to ameliorate CIRI [16]. These findings highlight the importance of microglial polarization in CIRI and suggest that M1 polarization of microglia acts as a risk factor for exacerbating the condition, whereas M2 polarization offers a protective and ameliorative effect. These observations align with our data, which show increased M1 polarization of microglia following CIRI. Whole transcriptome sequencing of ischemic brain tissue from the reperfused hemisphere in mice identified differentially expressed genes, revealing an upregulation of the deubiquitinating enzyme TNFAIP3 post-CIRI. Western blot analysis confirmed a significant increase in TNFAIP3 expression following CIRI compared to the sham-operated group, with the most pronounced elevation observed at 6 hours, after which TNFAIP3 levels declined. These findings suggest that TNFAIP3 may play a role in regulating the M1/M2 polarization of microglia and contribute to the worsening or amelioration of CIRI. Previous studies have shown that TNFAIP3 activates the Hippo-YAP signaling pathway, which is involved in mitigating hepatic ischemia-reperfusion injury [27]. Furthermore, Ginsenoside Rb1 has been shown to attenuate CIRI-induced brain injury by modulating the Nrf2/TNFAIP3/eEF1A2 axis [28]. Taken together, these findings suggest that the early elevation of TNFAIP3 within 6 hours post-I/R in our study likely represents a compensatory response to injury, aimed at exerting protective effects. The subsequent decline in expression may result from injury surpassing the reparative capacity of TNFAIP3, leading to extensive cellular damage.

Notably, ubiquitination plays a critical role in the initiation and progression of CIRI by regulating the expression of key genes. Chen et al. demonstrated that the deubiquitinating enzyme USP30 alleviates CIRI by preventing mitochondrial degradation, which occurs through the inhibition of mitofusin 2 (MFN2) ubiquitination [29]. Huang et al. reported that the long non-coding RNA (lncRNA) SNHG3 mitigates CIRI by inhibiting microglial activation via suppression of HDAC3 ubiquitination [30]. Ubiquitination has also been shown to exacerbate CIRI by modulating the expression of critical genes. Xia et al. illustrated that TRIM45 regulates microglia-mediated neuroinflammation by promoting the polyubiquitination of TAB2 at K63, thereby intensifying CIRI [31]. Similarly, Gao et al. demonstrated that the E3 ubiquitin ligase FBXO3 exacerbates CIRI by inducing the proteasomal degradation of HIPK2 through ubiquitination [32]. TNFAIP3, an enzyme involved in ubiquitination, has been implicated in the prognosis of various diseases. Research indicates that TNFAIP3 participates in neuroinflammation and suppresses necrotic cell apoptosis by modulating the expression of ICOSL [33], TRAF6 ubiquitination [34], and RIPK3 kinase ubiquitination [35]. Notably, prior studies have shown that upregulation of TNFAIP3 negatively regulates necrosis and M1 polarization of microglia/macrophages by inhibiting RIP3 ubiquitination, thereby improving CIRI [36]. In contrast, our study employed an in vitro OGD/R model to identify RACK1 as a novel ubiquitinated substrate of TNFAIP3A. Furthermore, a separate study demonstrated that rosmarinic acid (RA) inhibits lipopolysaccharide (LPS)-induced or sepsis-induced microglial M1 polarization in sepsis-surviving mice via the RACK1/HIF-1α pathway [37]. Similarly, our findings confirm that TNFAIP3 suppresses M1 polarization in microglia and promotes CIRI improvement by inhibiting the ubiquitination of RACK1.

RACK1 is a conserved WD40 repeat scaffold protein that functions as a versatile signaling adaptor. It is involved in various biological processes, including cancer progression [38], neural development [39], and virus replication [40]. Li et al. found that RACK1 overexpression reduces infarct size, neuronal death, tissue loss, and neurobehavioral dysfunction [41], which aligns with our results demonstrating that RACK1 overexpression improves CIRI. Our research suggests that this improvement is mediated by RACK1-induced M2 polarization of microglia. Zhao et al. demonstrated that RACK1 expression increases following CIRI, providing neuroprotection via the PINK1/Parkin pathway [42]. Li et al. reported that following CIRI, RACK1 expression initially decreases between 1.5 and 4 hours, gradually rebounding thereafter [41]. This observation contrasts with our findings, where RACK1 expression decreased after CIRI and remained low for 6 hours before rebounding. We speculate that this discrepancy may stem from differences in the MCAO/R model used. Consistent with previous studies, our results also show an increase in RACK1 expression in rat brain tissue 24 hours post-MCAO/R surgery [42], and we observe elevated RACK1 levels at both 24 and 48 hours post-surgery.

Recent research has revealed that TNFAIP3 functions as a cytoplasmic ubiquitin ligase and serves as a key negative regulator of the NF-κB signaling pathway [43]. By modulating the ubiquitinated status of factors such as NF-κB and IRF3, TNFAIP3 plays a central role in regulating immunity and inflammation [44,45]. Ji et al. demonstrated that VSIG4 attenuates neuroinflammation following cerebral hemorrhage by upregulating TNFAIP3 expression [46]. Electroacupuncture has been shown to mitigate CIRI by upregulating TNFAIP3 expression in neurons, thereby regulating the NF-κB pathway [47]. Furthermore, the lncRNA MEG3 inhibits microglial M1 polarization in acute spinal cord injury (ACI) through the HuR/A20/NF-κB axis [48]. In a similar vein, our study reveals that TNFAIP3 modulates microglial polarization following CIRI and contributes to disease improvement. In contrast to previous studies, our findings indicate that RACK1 serves as a critical intermediary factor in this process. Specifically, TNFAIP3 exerts a positive effect on CIRI improvement by inhibiting M1 polarization of microglia through the deubiquitination of RACK1. Moreover, RACK1 overexpression was found to prevent neuronal injury induced by CIRI via the PINK1/Parkin pathway [42]. These findings suggest that, in addition to ubiquitination, NF-κB-mediated inflammatory responses may also play a significant role. We confirmed that RACK1 exerts its neuroprotective effects through NF-κB, as demonstrated by the use of NF-κB inhibitors.

**Fig 8:**
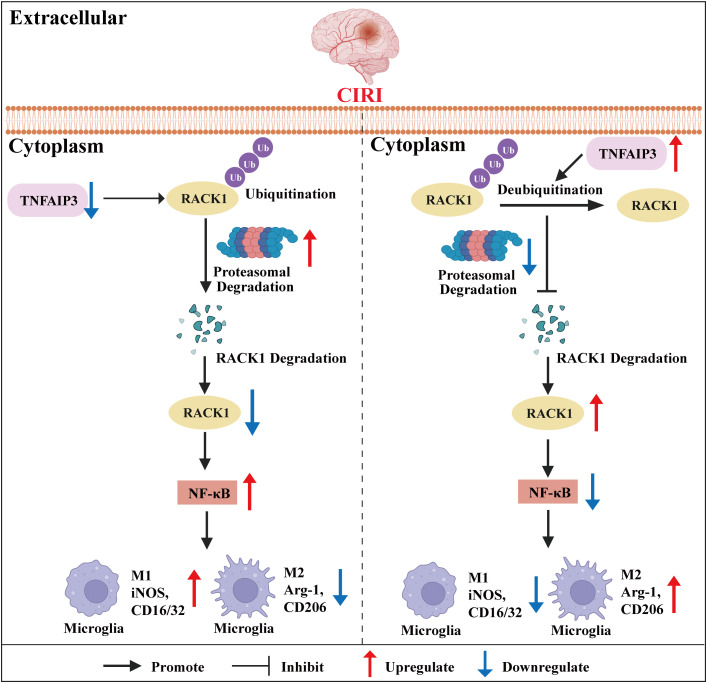
Mechanistic diagram illustrating how TNFAIP3 alleviates cerebral ischemia-reperfusion injury by inhibiting microglial M1 polarization via the deubiquitination of RACK1.

Although our study elucidates the mechanism by which TNFAIP3 inhibits M1 polarization of microglia and NF-κB-mediated inflammatory responses through the deubiquitination of RACK1 (Fig 8), several limitations remain. Site-directed mutagenesis of key lysine residues on RACK1 to identify K48 ubiquitin chains, rescue assays with RACK1 mutants exhibiting defects in deubiquitination or phosphorylation, and the identification of regulated targets through transcriptomic or phosphoproteomic analysis are crucial steps for confirming the causality of our findings. These additional experiments will provide compelling evidence to support our conclusions. Moreover, the efficacy of reperfusion varies across multiple subtypes of acute ischemic stroke. Whether our findings can partially explain the underlying reasons warrants further investigation and discussion. Furthermore, the specific molecular mechanisms underlying TNFAIP3-mediated deubiquitination of RACK1 require further clarification. Finally, as our study is based primarily on male animal models and in vitro cell experiments, additional validation in clinical settings and consideration of potential sex differences are warranted.

## Conclusion

In conclusion, the current study elucidated the mechanism of CIRI in the TNFAIP3/RACK1/ NF-κB signaling axis from the perspective of the post-translational modification of proteins, and established a theoretical foundation for developing strategies to prevent and control CIRI by targeting TNFAIP3 and RACK1 to regulate microglial polarization.

## Supporting information

S1 File WB-not for publication. (DOCX)

S2 File Total raw data. (XLSX)
