# Supplementary material for: TNFAIP3 alleviates cerebral ischemia-reperfusion injury by inhibiting M1 microglia polarization via deubiquitination of RACK1
Source: PLoS One. 2025 Nov 26;20(11):e0337601. doi: 10.1371/journal.pone.0337601 (PMC12654896; doi:10.1371/journal.pone.0337601)
Supplement: S1 File — (DOCX) [file pone.0337601.s001.docx]

Marker: Prestained Protein Marker IV


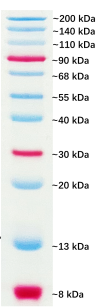


Figure 1

β-actin





TNFAIP3





INOS





Arg-1





Figure 2

F2C

β-actin





RACK1





F2D

β-actin





RACK1





F2E

β-actin





RACK1





Figure 3

β-actin





INOS





CD16/32





Arg1





CD206





Figure 5

β-actin





INOS





CD16/32





Arg1





CD206





Figure 7

β-actin





INOS





CD16/32





Arg1





CD206
